# Trend and spatial pattern of intimate partner rape notifications against women in Northeast Brazil (2013–2022)

**DOI:** 10.1590/1980-549720240030

**Published:** 2024-06-14

**Authors:** Marília Ramalho Oliveira, Alberto Pereira Madeiro, Fernando Ferraz Nascimento, Jesusmar Ximenes Andrade, Malvina Thais Pacheco Rodrigues, Márcio Dênis Medeiros Mascarenhas

**Affiliations:** I Universidade Federal do Piauí – Teresina (PI), Brazil.

**Keywords:** Violence against women, Intimate partner violence, Sexual violence, Rape, Northeast

## Abstract

**Objective:**

To analyze the trend and spatial pattern of intimate partner rape reports against women in Northeast Brazil.

**Methods:**

Ecological time-series study and spatial analysis with secondary data from the Notifiable Diseases Information System between 2013 and 2022. Gross rape rates were calculated by type of intimate partner and by age group of the victim. Prais-Winsten regression was used to calculate the trend, and the global and local Moran indices were used for spatial analysis.

**Results:**

A total of 5,542 cases of intimate partner rape were reported. Spousal rates ranged from 0.34/100,000 women in 2013 to 0.51/100,000 in 2017, with greater increases between 2018 (1.04/100 thousand) and 2022 (1.28/100 thousand). There was an upward trend in the Northeast as a whole (APC=19.47; 95%CI 15.88–23.22) and in almost all states, except Paraíba and Sergipe. Cases perpetrated by boyfriends (APC=23.90; 95%CI 12.80–36.09) and among women aged 15 to 19 years (APC=22.63; 95%CI 4.18–44.35) showed the highest annual variation. A concentration of high rates was observed in several municipalities in the northwest of Ceará and southeast of Pernambuco.

**Conclusion:**

The trend in intimate partner rape rates against women increased in the Northeast, especially among younger women and by boyfriends, with a greater agglomeration of notifications in Ceará and Pernambuco.

## INTRODUCTION

Intimate partner violence against women is a significant public health and human rights concern globally, as it profoundly impacts the lives, physical well-being, and health of women^1,2^. Statistics indicate that approximately 1 in 3 women have experienced physical and/or sexual violence at some stage in their lives, often perpetrated by an intimate partner^3^. Intimate partner violence encompasses various forms of harmful behavior within marital relationships — and may also be perpetrated by a former partner —, including physical, psychological, sexual, emotional, economic, and coercive control tactics^1-4^.

Sexual violence, a prevalent form of intimate partner violence, encompasses any sexual contact obtained without consent^2,5,6^. Among its various manifestations, rape is considered the most severe. In accordance with Brazilian law, rape is defined as coercing or compelling someone, through serious threats or physical force, to engage in sexual intercourse or to perform or endure any other sexual act^7^. Similar to the repercussions of sexual violence perpetrated by unknown assailants, intimate partner sexual violence inflicts serious consequences on women's health, including depression, substance abuse, and suicidal ideation^8^.

Data from 161 countries in 2018 showed that 27% of women aged 15 to 49 have experienced physical or sexual violence (or both) caused by an intimate partner throughout their lives^1^. Furthermore, 38 to 50% of all murders of women in the world are committed by partners or ex-partners^9^. In Brazil, intimate partners were responsible for 62.4% of reported violence against women between 2011 and 2017, with sexual violence disproportionately affecting pregnant women and individuals with disabilities^10^. Moreover, during the COVID-19 pandemic (March 2020 to December 2021), over 100,000 cases of rape against women were documented, with 18 Brazilian states surpassing pre-pandemic levels, particularly in the Northeast region (Paraíba, Maranhão, Alagoas, Piauí, Sergipe, and Rio Grande do Norte)^11^.

Indeed, there is a lack of studies specifically addressing rape by intimate partners. Although it is known that intimate partners are more likely to perpetrate some type of sexual violence against their partners or ex-partners^1,5,6^, this type of aggression may be less identified by women and, consequently, underreported. Many, for example, may not have internalized one of the most common stereotypes about rape: that it can occur within an intimate relationship^1,2^.

Furthermore, the Northeast region has recently been identified as having the highest percentage of exposure to violence among adults^12^ and a place where many women still find themselves in a patriarchal regime with great stratification of gender, social status, and race^13^. Based on this data, this study aimed to analyze the trend and spatial pattern of notifications of rape by an intimate partner against women in the Northeast.

## METHODS

This study adopted an ecological approach to conduct temporal and spatial analysis, focusing on the examination of publicly available secondary data. These data were sourced from the Notifiable Diseases Information System (*Sistema de Informação de Agravos de Notificação* – SINAN) within the Information Technology Department of the Unified Health System (*Departamento de Informática do Sistema Único de Saúde* – DATASUS). All information was filtered according to the following categories: Northeast region, female gender, age range >15 years, rape (yes), and intimate partner (boyfriend, ex-boyfriend, spouse, and ex-spouse). The variables of interest were considered: year of notification, state of occurrence, municipality of occurrence, race/skin color, education, place of occurrence, repeated violence and suspected alcohol use.

The year of the reported rape incidents (spanning from 2013 to 2022) was treated as the independent variable, while the crude rate of intimate partner rape served as the dependent variable, categorized by state, age range, and type of intimate partner. Crude rates of intimate partner rape were computed by dividing the number of reported rape cases involving women aged 15 years old and older by an intimate partner, by the corresponding population of women aged 15 years old and older within the same location and time frame. Intercensal estimates spanning from 2013 to 2022 provided by the Brazilian Institute of Geography and Statistics (*Instituto Brasileiro de Geografia e Estatística* – IBGE) were utilized for this calculation^14^.

A descriptive analysis was conducted, presenting absolute frequencies (n), relative frequencies (%), mean (M), and standard deviation (SD). To assess the temporal trend, Prais-Winsten regression was employed. Logarithmic transformation of the dependent variables to base 10 logarithms was performed to address issues of variance heterogeneity and facilitate the calculation of the annual percentage change (APC) along with its corresponding 95% confidence interval (95%CI)^15^. A positive APC, in conjunction with a statistically significant regression model (p<0.05), suggests an increasing trend. Conversely, a negative APC, alongside a statistically significant regression model (p<0.05), indicates a decreasing trend. A stationary trend is inferred if the regression model lacks statistical significance (p>0.05). Durbin-Watson values (corrected by Prais-Winsten) were computed to identify the presence of first or second-order autocorrelation (results falling between 1.5 and 2.5 are considered conducive for trend interpretation).

For the spatial analysis, the univariate Moran Global and Local Index (GMI/LMI) was utilized. The entire northeastern macro-region served as the geographical scope for this model, with municipalities acting as the units of analysis and the rape rate as the focal event. The map was derived from the IBGE cartographic base (version 2020).

The Moran Index assesses the presence of spatial dependence, indicating whether the event of interest exhibits discernible spatial patterns on the map. The Global Moran Index (GMI) values range from -1 to +1, where positive values (between 0 and +1) denote direct correlation (high-high or low-low), while negative values (between 0 and -1) signify inverse correlation (high-low or low-high). Statistically significant results indicate the presence of overall spatial dependence on the map. However, GMI does not pinpoint where these dependency patterns occur. Therefore, the Local Moran Index (LMI) was employed to identify clusters within each municipality^16^.

For mapping the data, Bayesian Empirical Spatial Rates (BESR) were utilized, deemed most suitable for generating maps that display significant regional disparities or encompass numerous polygons. BESR takes into account the neighborhood matrix (local average of neighboring municipalities)^17^. This rate was computed using the total reported rape cases from 2013 to 2022, alongside the corresponding population figures obtained from the IBGE Census^14^. The model parameters included the cartographic projection in the Geocentric Reference System for the Americas 2000 (SIRGAS 2000) and the queen contiguity neighborhood matrix with first-order neighbors. Municipalities with no reported rape incidents were excluded from the analysis.

To assess the significance of all generated models (GMI or LMI), the pseudo-significance test was conducted using 99,999 permutations^16^. The cartographic representation was achieved using the Local Indicators of Spatial Association (LISA) map, which categorizes municipalities based on LMI. The time series analysis was performed using Stata (version 17). GMI/LMI and BESR were executed in GeoDa (version 1.20), and the maps were generated in QGIS 4.24 Tisler. A significance level of 5% (p<0.05) was adopted for trend estimates, while a significance level of 1% (p<0.01) was applied for maps to address the issue of multiple comparisons (99,999 permutations).

This study was not submitted for consideration by the Research Ethics Committee because it involves secondary data, as per Resolution No. 510/2016 of the National Health Council^18^.

## RESULTS

5,542 cases of intimate partner rape against women were reported. The highest number of notifications occurred in 2022 (18.9%), while the lowest was recorded in 2014 (4.0%). Table 1 reveals that throughout the period, Pernambuco had the highest number of notifications (42.4%), followed by Bahia (17.9%). Most victims were up to 39 years old (76.5%), with nearly a quarter of them (23.3%) being teenagers. Additionally, there was a higher frequency of individuals with brown race/skin color (63.1%), with education up to elementary school (39.9%). Violence was primarily perpetrated by spouses (41.3%) and ex-spouses (31.0%), occurring predominantly at home (82.5%), repeatedly (71.8%), and without the aggressor having consumed alcoholic beverages (48.2%).

**Table 1:** Number of reports of rapes against women perpetrated by intimate partners. Northeast Brazil, 2013-2022.

Characteristics (n=5,542)		N	%
State			
	Alagoas	179	3.2
	Bahia	993	17.9
	Ceará	900	16.2
	Maranhão	279	5.0
	Paraíba	355	6.4
	Pernambuco	2,351	42.4
	Piauí	314	5.7
	Rio Grande do Norte	87	1.6
	Sergipe	84	1.5
Relationship with the perpetrator			
	Spouse	2,137	41.3
	Ex-spouse	1,603	31.0
	Boyfriend	818	15.8
	Ex-boyfriend	621	12.0
Age range of the victim (in years)			
	15–19	1,292	23.3
	20–29	1,396	25.2
	30–39	1,537	27.7
	40–49	893	16.2
	50–59	309	5.6
	60 or more	112	2.0
Race/skin color*			
	White	1,004	18.1
	Black	781	14.1
	Yellow	61	1.1
	Brown	3,497	63.1
	Indigenous	27	0.5
Education†			
	Illiterate	87	1.6
	(Incomplete/complete) elementary education	1,657	39.9
	(Incomplete/complete) high school education	1,950	25.2
	(Incomplete/complete) higher education	719	13.0
Location of occurrence			
	Residence	4,571	82.5
	Public places	263	4.7
	Others‡	12	12.8
Recurrent violence§			
	Yes	3,978	71.8
	No	1,208	21.8
Suspected alcohol use//			
	Yes	2,027	36.6
	No	2,672	48.2

* Ignored/blank: 172 notifications (3.1%);

† Ignored/blank: 1,129 notifications (20.4%);

‡ Others: Collective housing/school/sports venue/bar or similar/commerce/services/industries/construction/ignored/blank;

§ Ignored/blank: 356 notifications (6.4%);

// Ignored/blank: 843 notifications (15.2%).

Source: Adapted by the authors according to data from the Notifiable Diseases Information System (*Sistema de Informação de Agravos de Notificação* – SINAN), 2023.

There was a gradual increase in crude rape notification rates for all types of intimate partners, with higher rates observed for spouses. Between 2013 and 2017, the rates perpetrated by spouses ranged from 0.34 per 100 thousand women in 2013 to 0.51 per 100 thousand in 2017, with more significant increases noted between 2018 (1.04 per 100 thousand) and 2022 (1.28 per 100 thousand). Regarding other intimate partners, there was a slight increase in rates during the period, except for rape committed by a boyfriend, where the year 2022 exhibited the highest notification rates in the time series (Table 2).

**Table 2:** Gross rates of rape against women, according to the type of intimate partner. Northeast, Brazil, 2013-2022.

Year	Spouse			Ex-spouse			Boyfriend			Ex-boyfriend			Total		
	n	%	GR^a^	n	%	GR^a^	n	%	GR	n	%	GR^a^	n	%	GR*
2013	95	4.4	0.34	60	3.7	0.20	37	4.5	0.13	22	3.5	0.08	243	4.4	0.86
2014	103	4.8	0.36	61	3.8	0.20	20	2.4	0.07	20	3.2	0.07	223	4.0	0.78
2015	96	4.5	0.33	90	5.6	0.30	34	4.2	0.12	27	4.3	0.09	270	4.9	0.94
2016	133	6.2	0.46	111	6.9	0.38	33	4.0	0.11	44	7.1	0.15	347	6.3	1.20
2017	147	6.9	0.51	147	9.2	0.51	49	6.0	0.17	55	8.9	0.19	441	8.0	1.52
2018	305	14.3	1.04	155	9.7	0.53	90	11.0	0.31	69	11.1	0.24	652	11.8	2.23
2019	283	13.2	0.96	161	10.0	0.55	105	12.8	0.36	74	11.9	0.25	668	12.1	2.27
2020	242	11.3	0.82	164	10.2	0.55	184	22.5	0.62	63	10.1	0.21	691	12.5	2.34
2021	349	16.3	1.17	289	18.0	0.97	156	19.1	0.52	110	17.7	0.37	958	17.3	3.22
2022	384	18.0	1.28	365	22.8	1.22	110	13.4	0.37	137	22.1	0.46	1,049	18.9	3.51
**Total**	2,137	100	–	1,603	100	–	818	100	–	621	100	–	5,542	100	–
Mean†	–	–	0.73	–	–	0.55	–	–	0.28	–	–	0.21	–	–	1.89
SD	–	–	0.37	–	–	0.32	–	–	0.18	–	–	0.12	–	–	0.98

* Gross rape rate per 100,000 women;

† Mean annual crude rate of intimate partner rape. SD: Standard deviation.

Source: Adapted by the authors according to data from the Notifiable Diseases Information System (*Sistema de Informação de Agravos de Notificação* – SINAN), 2023.

Table 3 presents the temporal trend in crude rape rates. Among the states, only Paraíba (APC = -0.35; 95%CI -15.81–17.94) and Sergipe (APC = 12.65; 95%CI -14.92–49.15) exhibited a stationary trend, while the remaining states showed an increasing trend. Ceará had the highest percentage of annual growth (APC = 42.11; 95%CI 22.46–64.91), followed by Alagoas (APC = 34.51; 95%CI 20.91–49.63), with Piauí showing the lowest annual growth (APC = 14.40; 95%CI 1.94–28.38). Concerning the main aggressor, an increase was observed for all types, with cases perpetrated by boyfriends (APC = 23.90; 95%CI 12.80–36.09) and ex-boyfriends (APC = 22.39; 95%CI 16.40–28.69) exhibiting the greatest annual variation. Regarding the age range, a higher increase in rape rates was noted among women aged 15 to 19 (APC = 22.63; 95%CI 4.18–44.35).

**Table 3:** Trend in gross rape rate per 100,000 women, according to Northeastern states, main aggressor and age group of the victim. Northeast, Brazil, 2013-2022.

Characteristics		APC (%)	95%CI	p-value	DW	Trend
State						
	Alagoas	34.51	20.91; 49.63	<0.001	1.964	Growing
	Bahia	20.23	12.12; 28.94	<0.001	1.735	Growing
	Ceará	42.11	22.46; 64.91	0.001	1.433	Growing
	Maranhão	19.58	8.85; 31.37	0.002	1.643	Growing
	Paraíba	-0.35	-15.81; 17.94	0.963	1.791	Stationary
	Pernambuco	18.39	10.22; 27.17	0.001	1.854	Growing
	Piauí	14.40	1.94; 28.38	0.027	1.525	Growing
	Rio Grande do Norte	23.87	7.38; 42.89	0.009	1.962	Growing
	Sergipe	12.65	-14.92; 49.15	0.356	1.543	Stationary
	All	19.47	15.84; 23.22	<0.001	1.782	Growing
Relationship with the perpetrator						
	Spouse	18.31	12.51; 24.39	<0.001	1.955	Growing
	Ex-spouse	20.31	15.23; 25.61	<0.001	1.786	Growing
	Boyfriend	23.90	12.80; 36.09	0.001	1.607	Growing
	Ex-boyfriend	22.39	16.40; 28.69	<0.001	1.871	Growing
Age range (in years)						
	15-19	22.63	4.18; 44.35	0.020	1.595	Growing
	20-29	18.68	15.45; 22.00	<0.001	1.834	Growing
	30-39	17.31	12.76; 22.05	<0.001	1.746	Growing
	40-49	17.88	13.98; 21.92	<0.001	1.843	Growing
	50-59	17.81	10.84; 25.21	<0.001	1.542	Growing
	60 or more	11.82	6.45; 17.46	0.001	1.911	Growing

APC: annual percentage change; 95%CI: 95% confidence interval; DW: Durbin-Watson corrected by the Prais-Winsten technique.

Source: Adapted by the authors according to data from the Notifiable Diseases Information System (*Sistema de Informação de Agravos de Notificação* – SINAN), 2023.

**Figure 1:**
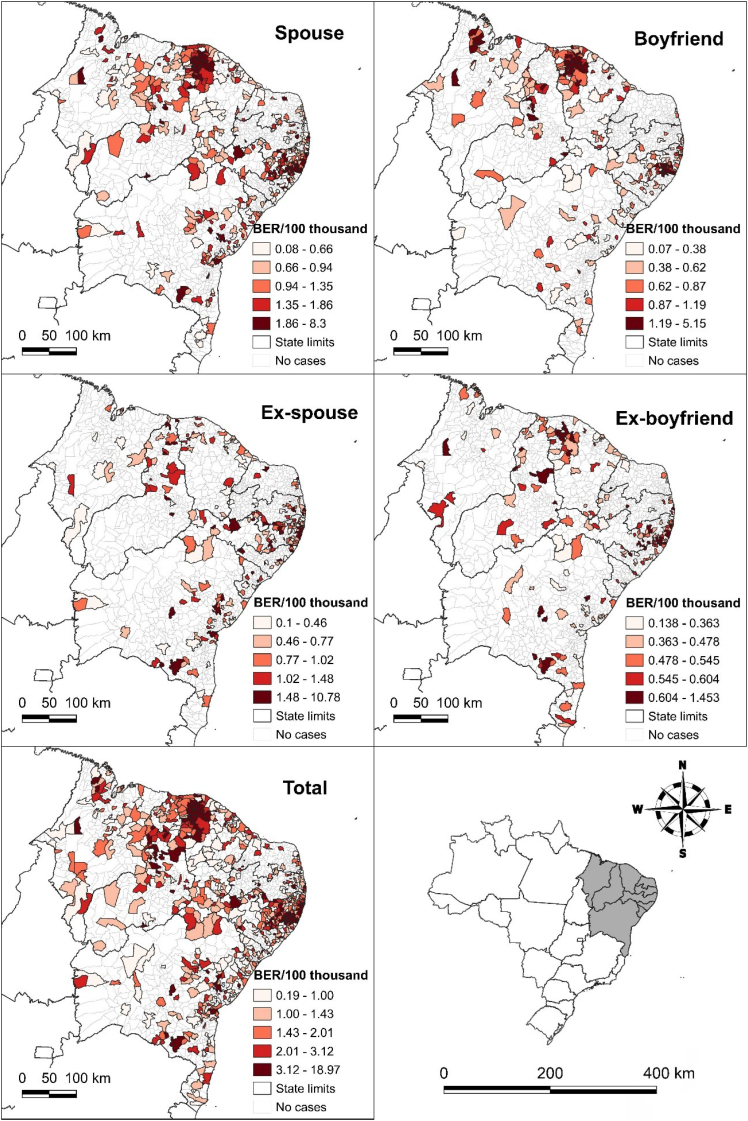
Spatial distribution of the empirical Bayesian rate of rape per 100 thousand women, according to municipalities and northeastern states and main aggressor, Northeast, 2013-2022.

Figure 1 illustrates the spatial distribution of Bayesian rape rates. A concentration of high rates is observed in several municipalities located in the states of Ceará and Pernambuco. This pattern is evident in both total rates and incidents caused by spouses and boyfriends, showing a higher concentration of rape rates. The global spatial dependence analysis (GMI) indicated that total rape rates, as well as those perpetrated by spouses (I=0.333), boyfriends (I=0.472), and ex-boyfriends (I=0.308), exhibited direct spatial autocorrelation (p<0.001). However, occurrences by ex-spouses were not statistically significant (p=0.108).

BER: Bayesian Empirical Rate per 100 thousand women.

Source: Adapted by the authors according to data from the Notifiable Diseases Information System (*Sistema de Informação de Agravos de Notificação* – SINAN), 2023

**Figure 2:**
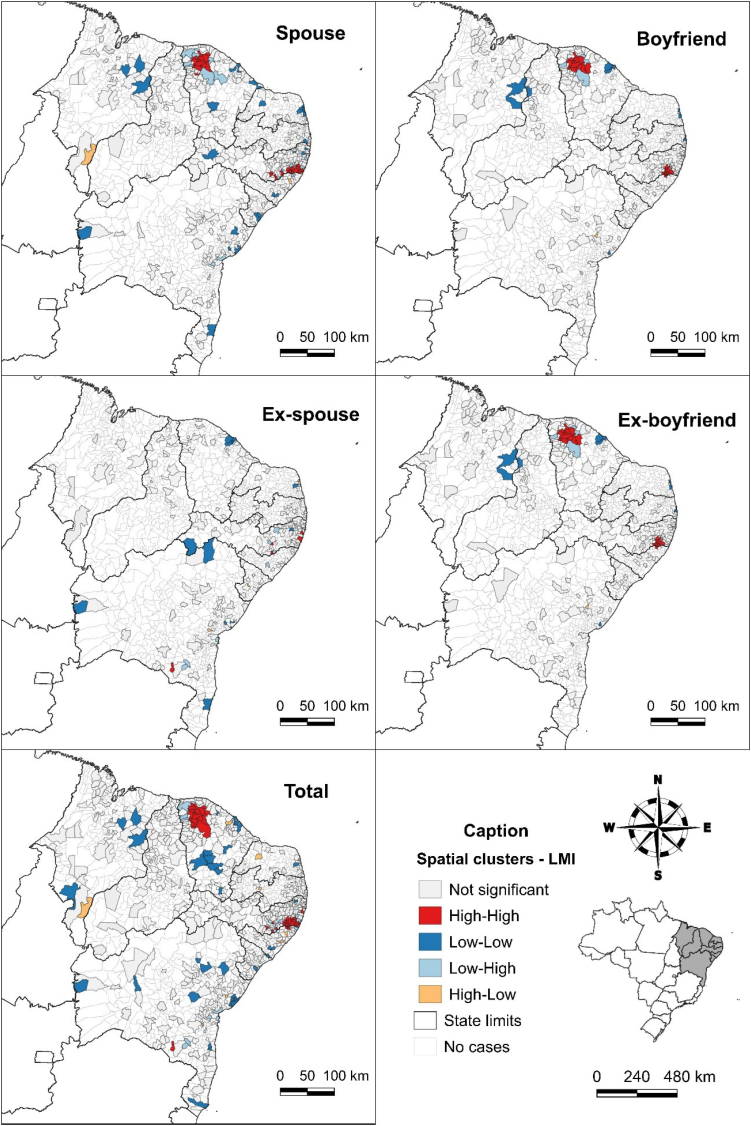
Spatial clusters of the Bayesian rape rate per 100 thousand women, according to northeastern municipalities and states and main aggressor, Northeast, Brazil, 2013-2022.

Figure 2 displays the results of Moran's local indices, categorized by types of aggressors. Spatial clusters of high Bayesian rape rates were identified in several municipalities in Ceará and Pernambuco. With the exception of cases of rape perpetrated by ex-spouses, the clusters are located in municipalities in the northwest of Ceará and the southeast of Pernambuco, near the border with Alagoas.

LMI: Local Moran Index.

Source: Adapted by the authors according to data from the Notifiable Diseases Information System (*Sistema de Informação de Agravos de Notificação* – SINAN), 2023.

## DISCUSSION

The notification profile revealed a predominance of young, brown women experiencing violence in their own homes, primarily by their spouses and without reported alcohol involvement. There was a notable trend of increasing crude rape rates across almost all states, involving various types of intimate partners and victim age groups. Additionally, the spatial analysis unveiled clustering of cases in Pernambuco and Ceará. These findings underscore that intimate partner rape, occurring within marital partnerships^1,19,20^, constitutes a particularly demeaning form of violence, as it is perpetrated by someone with whom the victim shares close emotional ties, often in what should be a safe environment^1,8,21^.

The concentration of notifications among women up to the age of 39 aligns with findings from previous studies on intimate partner violence in general^11,19,22,23^. For instance, data from 86 urgent and emergency services across 25 Brazilian capitals in 2014 indicated that 65% of women subjected to intimate partner rape were aged between 20 and 30 years old. Similarly, a survey spanning nine countries from 2000 to 2004 revealed that adolescents and young adults are particularly vulnerable to experiencing physical and/or sexual violence from an intimate partner, with this risk decreasing as individuals age^24^. Risk factors commonly associated with such violence include alcohol consumption and controlling behaviors by the partner, while higher levels of education and formal marriage are considered protective factors^1,24^.

Although influenced by various factors such as low socioeconomic status and unemployment, lower levels of education appear to correlate with higher rates of intimate partner violence^1,24,25^, as evidenced in this study. A population-based study conducted in Florianópolis (Santa Catarina) between 2009 and 2010 revealed that women with less than 5 years of education experience three times higher rates of moderate violence and five times higher rates of serious intimate partner violence compared to those with 5 or more years of education^26^. Additionally, among women with lower levels of education and income, incidents of sex forced out of fear or physical coercion were more prevalent^25^. Further research suggests an elevated risk of intimate partner violence when there exists an educational disparity between partners, particularly when the woman possesses a higher level of education^27^. It is plausible that increased education and financial independence may decrease women's tolerance for intimate partner violence^8,24^.

Consistent with the present study, prior research indicates that sexual violence predominantly occurs within current partnerships, whether defined as marital or boyfriend-girlfriend relationships, with over half of reported rape cases perpetrated by the spouse^19,20,22^. Nevertheless, marital status is strongly linked to intimate partner violence, as cohabiting women are more inclined to report instances of violence compared to women in formal marriages^26,27^.

In sub-Saharan African countries from 2010 to 2019, married women who exhibited greater sexual autonomy — manifested through their ability to refuse sex or request their partner to use a condom — were found to be at a heightened risk of experiencing sexual violence from an intimate partner^28^. This phenomenon can be understood within the context of deeply ingrained cultural norms that prioritize women's submission to men's desires. In such settings, husbands may perceive refusals or inquiries as a challenge to their authority, potentially leading to violent sexual acts against their partners^13,29^.

While intimate partner violence is acknowledged to transcend social classes, races, religions, educational levels, and even occur during pregnancy, there is consensus that certain factors can contribute to aggression, including alcohol intake^1,10,24,27,28,30^. Consumption of alcoholic beverages by an intimate partner heightens the likelihood of aggressive behavior, emotional abuse, and sexual misconduct^24,26,30,31^, with bidirectionality observed between substance abuse and marital violence^5,26^. Alcohol abuse affects both men and women, leading to diminished self-control and compromised cognitive function, thereby deteriorating marital relationships and exacerbating violent behavior^32^. However, data from the current research revealed a higher proportion of reported cases of rape without suspicion of alcohol use by partners. This finding warrants careful evaluation as the assessment of the partner's alcohol consumption is solely based on the woman's perception, and there is also a notable frequency of missing/blank responses on this topic.

The temporal trend analysis conducted in this study revealed an escalation in intimate partner rape rates across the Northeast region as a whole and in nearly all states. Although there are no national data available for comparison, a systematic review examining violence against women by intimate partners in the Americas identified a significant decrease in the lifetime prevalence of sexual violence in six countries (Colombia, Guatemala, Haiti, Mexico, Nicaragua, and Peru) out of the 24 evaluated during the period 1998–2018^20^. The observed increase in sexual violence trend in this investigation may partly be attributed to women's improved access to information and support from health and security services, which can facilitate the cessation of the cycle of violence and encourage reporting by victims to their intimate partners. However, it is also essential to consider other factors contributing to the perpetuation of intimate partner violence, including various social and economic vulnerabilities that constrain women's capacity to exit abusive relationships^19^.

Despite the observed increasing trend in rape rates perpetrated by all categories of intimate partners and across all age groups, it is notable that the highest percentage of annual growth occurred among cases involving boyfriends/ex-boyfriends and victims aged 15 to 19. These findings underscore the significance of violence within dating relationships, a phenomenon well-documented in other research^33,34^, where women are primarily victimized, particularly in cases of sexual and/or physical aggression. It is also important to highlight that this form of violence is increasingly facilitated by technology, such as the dissemination of sexual images and videos without consent^33,34^. A study examining sexual violence notification rates among adolescents revealed a rising trend across all regions of Brazil between 2011 and 2018, with the Northeast exhibiting the lowest rates and percentage variation^35^. Additionally, findings from a systematic review indicate that young women who cohabit with their partners are at even greater risk of experiencing violence, including sexual violence. This heightened vulnerability can be attributed to factors such as limited educational opportunities, power imbalances within relationships, and increased economic dependence, all of which constrain autonomy and predispose individuals to aggression^36^.

The study identified spatial heterogeneity in the crude notification rates of intimate partner rape in the Northeast, with heightened clusters observed in the states of Ceará and Pernambuco. Between 2009 and 2014, the majority of municipalities in Paraná that contributed to the formation of high-high clusters were associated with lower human development indices, a higher proportion of economically active women, and higher average female income^19^. On the one hand, apart from this geographic alignment with sociodemographic determinants, it is plausible that the promotion and perpetuation of violence are also intertwined with social and cultural norms surrounding ideals of masculinity and gender relations, which reinforce prevalent sexism in society, still pervasive in many locations in the Northeast^37^. However, it is recognized that women residing in smaller municipalities or rural contexts may encounter greater difficulty in reporting domestic violence or rape^38^.

Data from the World Health Organization (WHO) indicates that less than 40% of women who experience any form of violence seek assistance. The majority tend to rely on their support networks, primarily family and friends, while a smaller percentage turn to institutional resources such as health and security services (with less than 10% choosing to involve the police)^39^. Despite progress, the support network for women victims of violence in the Northeast remains limited. In 2022, only 10.7% of Police Stations Specialized in Women's Assistance operated 24 hours a day^40^, and in 2018, the region had 23.5% of the specialized service network and 23.8% of shelters for situations involving death threats^41^. A study conducted in Spain with 849 cases of sexual violence by an intimate partner revealed that common reasons for not reporting included the termination of the relationship, lack of awareness about reporting options, and expressions of shame, guilt, or fear undermining the credibility of the report^42^.

Finally, it is important to acknowledge several limitations of this study. Firstly, there may have been underreporting of rape, stemming from difficulties in women identifying violence and potential barriers to accessing healthcare facilities, as there remains an insufficient number of units providing specialized care for sexual violence, particularly in smaller municipalities^43^. Secondly, the comparison of rates and APC between states should be interpreted cautiously, considering the heterogeneity in data quality and significant variation in the completeness of various fields on the notification form^44,45^. Another limitation arises from the variables analyzed, which were constrained to those available in SINAN. Additional information common to the dynamics of intimate partner violence — such as whether there was overlap with physical and/or psychological aggression or whether the female victim was under the influence of alcohol/illegal drugs — would enhance understanding of the phenomenon. Furthermore, due to database constraints, it was not possible to select more than one answer for the type of intimate partnership. Given that revictimization and polyvictimization of women are common occurrences^2,3^, it was impossible to compare the prevalence of violence committed by the current/most recent partnership with the assessment of violence by any partner throughout life.

The findings of this study underscore the prevalence of sexual violence against women by intimate partners as a significant issue in the Northeast of Brazil. To attain a more comprehensive understanding of this phenomenon, future research should seek to obtain estimates in diverse sociocultural contexts. Moreover, it is imperative that health services tailored to women's health address this issue in a manner that fosters a sense of safety and encourages victims to come forward and report incidents. The observed growing trend across the region, encompassing all types of intimate partners and victim age groups, along with the identification of geographic areas of heightened vulnerability, underscores the ongoing necessity for sustained efforts to prevent and respond to such violence.
